# The biomedical discourse relation bank

**DOI:** 10.1186/1471-2105-12-188

**Published:** 2011-05-23

**Authors:** Rashmi Prasad, Susan McRoy, Nadya Frid, Aravind Joshi, Hong Yu

**Affiliations:** 1 Institute for Research in Cognitive Science, University of Pennsylvania, 3401 Walnut Street, Philadelphia, PA 19104, USA; 2 Department of Computer and Information Science, University of Pennsylvania, 3330 Walnut Street, Philadelphia, PA 19104, USA; 3 Department of Health Sciences, University of Wisconsin-Milwaukee, P.O. Box 413, Milwaukee, WI 53201, USA; 4 Department of Electrical Engineering and Computer Science, University of Wisconsin-Milwaukee, P.O. Box 784, Milwaukee, WI 53201, USA

## Abstract

**Background:**

Identification of discourse relations, such as causal and contrastive relations, between situations mentioned in text is an important task for biomedical text-mining. A biomedical text corpus annotated with discourse relations would be very useful for developing and evaluating methods for biomedical discourse processing. However, little effort has been made to develop such an annotated resource.

**Results:**

We have developed the Biomedical Discourse Relation Bank (BioDRB), in which we have annotated explicit and implicit discourse relations in 24 open-access full-text biomedical articles from the GENIA corpus. Guidelines for the annotation were adapted from the Penn Discourse TreeBank (PDTB), which has discourse relations annotated over open-domain news articles. We introduced new conventions and modifications to the sense classification. We report reliable inter-annotator agreement of over 80% for all sub-tasks. Experiments for identifying the sense of explicit discourse connectives show the connective itself as a highly reliable indicator for coarse sense classification (accuracy 90.9% and F1 score 0.89). These results are comparable to results obtained with the same classifier on the PDTB data. With more refined sense classification, there is degradation in performance (accuracy 69.2% and F1 score 0.28), mainly due to sparsity in the data. The size of the corpus was found to be sufficient for identifying the sense of explicit connectives, with classifier performance stabilizing at about 1900 training instances. Finally, the classifier performs poorly when trained on PDTB and tested on BioDRB (accuracy 54.5% and F1 score 0.57).

**Conclusion:**

Our work shows that discourse relations can be reliably annotated in biomedical text. Coarse sense disambiguation of explicit connectives can be done with high reliability by using just the connective as a feature, but more refined sense classification requires either richer features or more annotated data. The poor performance of a classifier trained in the open domain and tested in the biomedical domain suggests significant differences in the semantic usage of connectives across these domains, and provides robust evidence for a biomedical sublanguage for discourse and the need to develop a specialized biomedical discourse annotated corpus. The results of our cross-domain experiments are consistent with related work on identifying connectives in BioDRB.

## Background

Biomedical literature is a rich resource of biomedical knowledge. The desire to retrieve, organize, and extract biomedical knowledge from literature and then analyze the knowledge has boosted research in biomedical text mining. As described in recent reviews [1-4], the past 10 years have shown significant research developments in named entity recognition [5-7], relation extraction [8,9], information retrieval [10,11], hypothesis generation [12], summarization [13-16], multimedia [17-21], and question answering [22,23]. Garzone and Mercer [24,25] and Mercer and DiMarco [26] have explored how to connect a citing paper and the work cited. Light et al [27] have identified the use of speculative language in biomedical text. Wilbur et al. [28,29] defined five qualitative dimensions (i.e., *focus, polarity, certainty, evidence *and *directionality*) for categorizing the intention of a sentence.

Looking at larger units of text, Mullen et al. [30] and Yu et al. [20,31] defined discourse zones of biomedical text including *introduction, method, result*, and *conclusion*, and developed supervised machine-learning approaches to automatically classify a sentence into the rhetorical zone category. Biber and Jones [32] adapted unsupervised TextTiling methods [33] to segment biomedical text into different discourse units on the basis of lexical similarities among the units. "BioContrasts" [34] is an information extraction system that extracts contrastive information between proteins from texts on the basis of manually curated rules and regular expressions that focus on *negation *as an expression of contrast. Castano et al. [35] built a system for anaphora resolution in biomedical literature. Szarvas et al [36] annotated negation, speculation and scope in biomedical text. Agarwal and Yu [37,38] have investigated the detection of hedges, negation, and their scopes in biomedical literature.

One important output of this research on biomedical text has been the creation of new annotated resources specific to the biomedical domain. For example, the GENIA corpus is a collection of biomedical literature, annotated with various levels of linguistic and semantic information, including coreference [39]. The ART corpus [40,41] contains sentence-wise annotations of scientific papers (covering topics in physical chemistry and biochemistry) with core scientific concepts (e.g. *goal, hypothesis, experiment, method, result, conclusion, motivation, observation*). These resources are valuable because they can be used to evaluate the effectiveness of text-mining methods developed for the biomedical domain. They can also be used to evaluate whether methods developed for the open domain can generalize to biomedical literature, which then determines whether new biomedical-specific training data needs to be created.

To date, there has been little work on processing or annotating *discourse relations *in biomedical text. A *discourse *is considered to be a coherent sequence of clauses, sentences or propositions. *Discourse relations*, such as causal, temporal, and contrastive relations, are relations between eventualities and propositions mentioned in a text, from which we can draw deep or complex inferences about the text. Often, discourse relations are realized in text by explicit words and phrases, called *discourse connectives*, but they can also be implicit.

Many tasks, including question answering and information extraction, require one to retrieve and process information that spans more than a single sentence while also recognizing discourse relations that exist between sentences. For instance, in Example (1), queries related to the "conflicting interactions of MRL631 with γ-secretase" can only be answered accurately once the contrastive discourse relation, expressed with the connective *however*, between the two sentences is identified.

(1) Our studies suggest that MRL631 is not able to access intracellular *γ*-secretase for APP processing and APP traffoiking. However, it interacts with *γ*-secretase residing at the cell surface for Notch processing. From [42].

Causal and justification relations also constitute a very important part of the knowledge dealt with in information extraction, and are often expressed across sentences: for instance, the connective *therefore *in Example (2) signals a justification relation between the first two sentences, i.e, the fact that "there is the presence of a major 90-to-100-kDa protein of unknown sequence in both the rat otoconia and the Xenopus utricular (calcitic) otoconia" is the reason for believing that "calcitic otoconia contain a similar 90-to-100-kDa protein."

(2) In both the rat otoconia and the Xenopus utricular (calcitic) otoconia, the presence of a major 90-to 100-kDa protein of unknown sequence has been reported [3]. Therefore, calcitic otoconia probably contain a similar 90- to 100-kDa major protein, regardless of the species. In contrast, the Xenopus saccular (aragonitic) otoconia contain a major 22-kDa protein (otoconin-22) [5], which is a sPLA_2_-related 127-aa glycoprotein with two N-glycosylation sites. From [43].

Discourse relations can also be useful for categorizing citations and the relations between citations to enhance information retrieval: the connective *in contrast *in Example (2) signals a contrast relation between two cited articles, "3" and "5", mentioned in two different sentences.

Although the discourse relations in the examples above are explicitly expressed in the text by a discourse connective, this is not always the case. Discourse relations can also be implicit between sentences. In Example (3), for instance, a causal relation is inferred between the two sentences, i.e., "the overproduction of numerous cytokines in the synovial membrane" is inferred as being the result of "the membrane having an infiltrate of a variety of inflammatory cells." However, there is no explicit connective (e.g., *as a result*, or *so*) to express this relation.

(3) The synovial membrane of rheumatoid arthritis (RA) is characterized by an infiltrate of a variety of inflammatory cells, such as lymphocytes, macrophages, and dendritic cells, together with proliferation of synovial fibroblast-like cells. Numerous cytokines are overproduced in the inflamed joint.

The challenge of processing discourse relations involves several subtasks, which have been tackled in the open (non-specialized) domain.

*• Identifying discourse connectives*. Many of the lexical items that can function as explicit connectives also have other non-connective functions [44,45]. Thus, connectives need to be functionally disambiguated.

*• Identifying the arguments of discourse connectives*. In addition to identifying the connectives themselves, it is also important to accurately identify the two situations (called *arguments*) that the connectives relate, since they are not necessarily adjacent to each other [46-50]).

*• Identifying the senses (i.e., semantics) of the relation*. While detecting the senses of explicit connectives has met with a good degree of success [44,51,52], owing to the observation that explicit connectives are not very ambiguous, implicit relations, on the other hand, have proved to be much more challenging [53-58].

*• Deriving Composite Discourse Structures*. Once the elementary relation structures (i.e., a relation and its two arguments) have been identified, the task of combining these elementary structures into more complex structures has important ramifications for tasks such as summarization [59].

The largest effort at annotating discourse relations is the Penn Discourse Treebank, or the PDTB [49], which contains annotations of discourse relations on the open-domain Wall Street Journal corpus [60]. To facilitate discourse processing research in the biomedical domain, we have adopted the PDTB framework to annotate discourse relations, their arguments, and their senses in biomedical literature. The corpus we have selected is a 24-article subset of the GENIA corpus [39], which is a collection of articles from the biomedical literature. It has been compiled and annotated within the scope of the GENIA project, and the 24 articles (with a total of approx. 112000 word tokens and approx. 5000 sentences) that form our **Biomedical Discourse Relation Bank (BioDRB) **have also been annotated for coreference relations and citation relations [61].

In this article, we describe our work towards the creation of the BioDRB. We show that the PDTB framework can be successfully adapted to the biomedical domain, and that discourse relations can be reliably annotated. We present classification experiments for sense disambiguation of explicit connectives, showing that the BioDRB sense classifier performs as well as the PDTB classifier. We also present experiments to show that the current size of the BioDRB corpus may be sufficient for this task. Finally, we explored whether NLP methods developed using the PDTB can be generalized to the biomedical domain. For the same task of explicit connective sense detection, we show that a classifier trained on the PDTB performs poorly on BioDRB. These results highlight the discourse-level differences between the open domain and the biomedical domain, and support the need for developing a specialized corpus of biomedical texts annotated with discourse relations. The results of our cross-domain experiments are consistent with our related work on identifying connectives in the BioDRB [45].

## Methods

For annotating discourse relations in biomedical literature, we adapted the annotation framework of the Penn Discourse TreeBank (PDTB) [49]. The PDTB http://www.seas.upenn.edu/~pdtb annotates the argument structure, semantics, and attribution of discourse relations and their arguments over the 1 million word Wall Street Journal portion of the Penn Treebank [60]. It follows a lexically-grounded approach to discourse structure [62,63]. A discourse relation is defined as a strictly binary, informational relation between abstract objects (AOs) mentioned in a text, such as events, states, and propositions [64]. By convention, the two AO arguments are called Arg1 and Arg2, with Arg2 as the argument syntactically bound to the connective, and Arg1 as the other argument. Discourse connectives are words or phrases used to express discourse relations in text, and in the PDTB, they are drawn from three well-defined syntactic classes: subordinating conjunctions (e.g., *because, when, since, although*), coordinating conjunctions (e.g., *but, or, nor*) and adverbials (e.g., *however, otherwise, then, as a result, for example*). Example (4) shows the causal connective *because *and its two arguments. (Throughout this paper, phrases expressing discourse relations are underlined, Arg1 appears in italics, Arg2 appears in boldface, and the sense is provided in parentheses at the end of the example.) Also annotated in the PDTB are implicit discourse relations between adjacent sentences, for which annotation involves insertion of a connective that best expresses the relation, and other explicit expressions (called *alternative lexicalizations*) of relations that do not belong to the pre-defined syntactic classes. For sense classification, a three-tier hierarchical scheme was developed for the PDTB, from which one or more labels are selected for each relation. Attribution, which is also annotated in the PDTB, is not handled currently in BioDRB.

(4) *She hasn't played any music *since**the earthquake hit**. (Temporal:Succession)

PDTB contains 100 distinct types of discourse connectives. Of the total 40,600 tokens in the corpus, 19053 are realized by explicit expressions, either connectives or alternative lexicalizations. Over the years, the PDTB research group has developed an effective set of discourse annotation tools, guidelines, work flows, and validation methodologies that we have used as a basis for our work.

The PTDB annotation framework has several important advantages over alternative approaches. First, the framework focuses on identifying individual relations and their arguments, which are important for text mining, while remaining neutral on the higher-level discourse organization. This is important because there is little agreement among researchers on the specification of the most descriptively adequate data structure for representing discourse [65]. The structures proposed so far range from tree structures (e.g., Rhetorical Structure Theory (RST) [66], Linguistic Discourse Model (LDM) [67], and RST-based binary trees [68] to more complex forms that incorporate multiple inheritance (D-LTAG [63] and Segmented Discourse Representation Theory (SDRT) [69]), to full-fledged graphs (Discourse Graphbank [70]). The PDTB is, therefore, a particularly attractive framework since it aims to remain neutral with respect to higher-level discourse organization, and instead focuses on annotating the more local discourse relations. Higher-level structures in this approach are left to "emerge" from the annotations of low-level relations. Some recent investigations on the combinatorial possibilities of discourse relations in the PDTB suggests that directed acyclic graphs (DAGs), and not trees, may be the most appropriate structural representation for discourse [71,72].

Second, discourse relations in the PDTB are lexically anchored, for both explicit and implicit connectives. In the latter case, annotators "insert" a connective expression to express the implicit relation, and then proceed to annotate the sense of the inserted connective. Such a lexically-grounded approach substantially increases the inter-annotator agreement [73], as confirmed in our pilot annotation study [74,75].

Finally, since its release, the PDTB has been successfully used by many researchers for both linguistic and computational studies [44,46-48,50-52,54-57,71,72,76-84], which shows that there is much to be gained from adopting this approach. The PDTB framework has also been adopted for discourse annotation in other languages (e.g., Turkish [85], Hindi [86,87], Chinese [88], Czech [89] and Italian [90]) as well as other domains such as conversational dialogues [90].

## Results and Discussion

### Biomedical Discourse Relation Bank: BioDRB

In the BioDRB, we have annotated all explicit and implicit discourse relations, the arguments of discourse relations, and the senses of discourse relations. In keeping with the theory-neutral approach of PDTB, we annotate only individual relations and do not attempt to show dependencies across relations. We have adapted the PDTB guidelines to better incorporate discourse-level features specific to biomedical texts. Here we present some salient aspects of the BioDRB annotation guidelines. Further details are provided in the complete documentation of the guidelines [91], available from http://spring.ims.uwm.edu/uploads/biodrb_guidelines.pdf

#### Discourse Relations and their Realization

Discourse relations in the BioDRB are first broadly classified in terms of their manner of realization. There are four types of relations:

(a) Relations realized by *Explicit discourse connectives*,

(b) *Implicit *relations,

(c) Relations realized by *alternatively lexicalized *expressions (AltLex),

(d) Absence of a discourse relation, or *No Relation *(NoRel).

**Explicit Discourse Connectives **are closed-class lexical items drawn from four well-defined syntactic classes: subordinating conjunctions (Example 5), coordinating conjunctions (Example 6), discourse adverbials (Example 7), and subordinators (Example 8). The syntactic classes themselves are not provided as part of the annotation, but were rather used to train the annotators to identify connectives. Arguments of explicit connectives can be identified within the same sentence as the connective, i.e., *intra-sententially *(Example 5,6,8) or in different sentences, i.e., *inter-sententially *(Example 7).

(5) Because**RA PBMC include several cell types in addition to T cells**, *some inflammatory cytokines released from macrophages and other lymphocytes might have affected the production of IL-17 from T cells*. (Cause:Reason)

(6) *IL-17 was also detected in the PBMC of patients with osteoarthritis*, but** their expression levels were much lower than those of RA PBMC**. (Concession:Contra-expectation)

(7) *IL-17 production by activated RA PBMC is completely or partly blocked in the presence of the NF-κB inhibitor pyrrolidine dithiocarbamate and the PI3K/Akt inhibitor wortmannin and LY294002, respectively*. However, **inhibition of activator protein-1 and extracellular signal-regulated kinase 1/2 did not affect IL-17 production**. (Contrast)

(8) Recent observations demonstrated that *IL-17 can also activate osteoclastic bone resorption *by** the induction of RANKL (receptor activator of nuclear factor ***κ***B [NF-***κ***B] ligand), which is involved in bony erosion in RA **[7]. (Purpose:Enablement)

Annotation of an explicit connective proceeds by first identifying and marking the connective text span, then identifying and annotating the text spans associated with its two arguments, and finally, labeling the sense of the relation. Thus, for Example (5), the following information is annotated:

• *Relation type: *Explicit

• *Connective span: *"Because"

• *Arg1 span: *"some inflammatory cytokines released from macrophages and other lymphocytes might have affected the production of IL-17 from T cells"

• *Arg2 span: *"RA PBMC include several cell types in addition to T cells"

• *Sense: *Cause:Reason

An important task in annotating explicit connectives involves determining whether or not the lexical item in question expresses a discourse relation, i.e., a relation between two abstract objects. Several lexical items that function as discourse connectives have other non-connective functions as well. For instance, *also *as a discourse connective is used to express the presence of two AO items in a list, as in Example (9). However, *also *can sometimes be used in a non-list sense, when it is used to imply that something has been "presupposed" [92], as in Example (10).

(9) *These data show that ITK is required for IL-2 production induced by SEB in vivo, and may regulate signals leading IL-2 production, in part by regulating phosphorylation of c-jun*. **The data **also** suggest that perturbing T cell activation pathways leading to IL-2 does not necessarily lead to improved responses to SEB toxicity**. (Conjunction)

(10) To determine whether CD123+ cells in synovial tissue were also nuclear RelB+, formalin-fixed tissue was double-stained for RelB and CD123 without hematoxylin counterstaining.

**Implicit Relations **are annotated inter-sententially between sentences not related by an explicit connective, and only within paragraphs. If a discourse relation is inferred between the sentences, the annotator must *insert *a connective that best expresses the inferred relation, then mark the arguments, and finally, assign a sense to the relation. Example (11) shows that the annotator perceived Arg2 as standing in contrast with Arg1, that there is no explicit connective to relate these two arguments, and that the annotator inserted *on the other hand *as the connective to express the inferred relation.

(11) *Expression of the brca1 mutant in a p21-null background caused little rescue of the cells in the thymus, but provided a recovery in the lymph nodes that was equivalent to that produced in the p53-null background*. Implicit = On the other hand

**Introduction of the brca1 gene in cells carrying an antiapoptotic Bcl2 transgene induced significant rescue of cells in the thymus, but produced little recovery of cells in peripheral (lymph node) compartments**. (Contrast)

For implicit relations, it is crucial that the annotator does not perceive any "redundancy" in the expression of the relation after inserting the connective. A redundancy effect would instead lead to the annotation of the AltLex relation type, discussed next.

**Alternative Lexicalizations (AltLex) **of relations are also annotated inter-sententially. They are identified when a discourse relation is inferred between sentences not related by an explicit connective, but insertion of a connective to express the implicit relation leads to "redundancy" in the expression of the relation. What such redundancy means is that the relation has in fact been lexicalized, but with an expression that cannot be syntactically classified as an explicit connective. For instance, in Example (12), the situation described by Arg2 is implicitly perceived to be a result of the situation in Arg1, but insertion of an implicit connective such as *as a result *clearly creates a redundancy. In such cases, the annotator must look for and annotate the "AltLex" expression. In this example, the AltLex is identified with the subject-verb sequence *These results suggest*. In the annotation, AltLex spans are always fully contained within Arg2 spans. In Example (12), for instance, the underlined AltLex span is also in boldface, showing that it is contained in the Arg2 span.

(12) *As shown in *Figure 3a,3b, *the intensity of IL-10R1 expression on CD4+ T cells was signicantly increased in RA patients compared with in healthy controls*.

**These results suggest that the intracellular signal transduction pathway of IL-10 may be impaired in CD4+ T cells of active RA**. (Cause:Claim)

Syntactically, AltLex expressions are open class lexical items that cannot be defined as explicit connectives [81]. In particular, while explicit connective expressions are fixed, or lexically invariant, AltLex expressions result from a more productive and compositional process. They often appear as subject-verb sequences (Example 12), although other syntactic patterns are found as well, such as prepositional phrases and verb phrases. Semantically, they are typically composed of two elements - one that denotes the relation, and the other that refers anaphorically to Arg1. In Example (12), the verb *suggest *denotes the relation, whereas the subject *These results *refers anaphorically to Arg1.

**No Relation (NoRel) **is the type assigned when a sentence does not appear to relate to any other sentence in the prior text. NoRel is annotated in only two specific cases. The first kind of NoRel is annotated within the "Abstract" section of the articles, some of which are partitioned into "Background", "Case Presentation", "Results", "Conclusion", etc. These "Abstract" sections are not separated by any paragraph boundary, but we treat them as such, and indicate these boundaries with the NoRel label. Example (13) illustrates one such NoRel annotation from the "Abstract" section of an article.

(13) Background: CC Chemokine Receptor 3 (CCR3), the major chemokine receptor expressed on eosinophils, binds promiscuously to several ligands including eotaxins 1, 2, and 3. (...) *It is therefore important to elucidate the molecular mechanisms regulating receptor expression*. Implicit = NoRel** Results: In order to define regions responsible for CCR3 transcription, a DNAse hypersensitive site was identified in the vicinity of exon 1**.

The second kind of NoRel was annotated for typological errors that led, for example, to some sentences being duplicated in the article. Since we didn't want to admit a non-semantic repetition relation, these were annotated as NoRel. Such cases are rare in the corpus.

For NoRel, Arg1 and Arg2 are, by convention, the immediately adjacent and complete sentences.

#### Arguments of Discourse Relations

The smallest syntactic unit for the realization of an AO argument of a discourse relation is a clause, tensed or non-tensed. Verb phrases can also be legal arguments when the connectives are not verb phrase conjunctions themselves. In addition, because we take discourse relations to hold between AOs, nominalizations are allowed (Example 14) as arguments, since they can denote events.

(14) *She was originally considered to be at high risk *due to** the familial occurrence of breast and other types of cancer**, (Cause:Reason)

There are no syntactic constraints on how many clauses or sentences an argument can contain. Semantically, however, arguments are required to be *minimal *in that "only as much should be selected as an argument as is necessary for interpreting the relation". Example (15) shows Arg1 as well as Arg2 spanning over multiple sentences for the AltLex generalization relation. However, for both Arg1 and Arg2, all the included sentences are necessary and sufficient because for the generalization relation in question, the specific details as well as the generalization of the details are distributed across exactly these multiple sentences.

(15) *We show here that mice lacking ITK have much reduced IL-2 production and T cell expansion in response to SEB in vitro and in vivo. We also show that SEB induced the activation of the JNK MAPK pathway in responding T cells in vivo, and that ITK null T cells were defective in the activation of this pathway in vivo. However, toxicity analysis indicated that both WT and ITK null animals were similarly affected by SEB exposure*. **Our data suggest that ITK is required for full IL-2 secretion following SEB exposure, and that this may be due to the regulation of the JNK pathway by ITK in vivo. However, reducing T cell signals does not necessarily lead to better physiological responses to SEB exposure**. (Restatement:Generalization)

Finally, except for NoRel, there are also no constraints on how far away a relation's Arg1 and Arg2 arguments can be from each other. That is, they need not be adjacent. Example (16) shows Arg1 and Arg2 in non-adjacent sentences for the explicit connective *However*. Unlike PDTB, where arguments of implicit relations are required to be adjacent, implicit relations in BioDRB can have non-adjacent arguments.

(16) The studies concerning the functional interaction between the NF-*κ*B pathway and members of the steroid hormone receptor family, and their role in synovial inflammation, have advanced significantly, *although with controversial results *[10,11]. In particular, after binding with E2, oestrogen receptors have been shown to interact with NF-*κ*B factors, via transcriptional co-factors, resulting in mutual or non-mutual antagonism. Other studies hypothesize that, since oestrogen receptors may repress both constitutive and inducible NF-*κ*B, the overexpression of NF-*κ*B-inducible genes in oestrogen receptor-negative cells might contribute to malignant cell growth and chemotherapeutic resistance [12,13]. On the contrary, further studies report that E2 blocks the transcriptional activity of p65 in macrophages [14]. However, **these opposite observations arise using different cell lines (human/animals) and culture conditions as well as different hormone concentrations **[15]. ...

#### Senses of Discourse Relations

All explicit, implicit and AltLex relations are annotated with sense labels that indicate their semantics. Senses are organized in two tiers, with the second *subtype *tier specifying further refinements to the sense *type *in the top tier. The complete BioDRB sense classification is shown in Table 1.

**Table 1:** BioDRB sense classification for discourse relations

Type	Subtype	Type	Subtype
CAUSE	Reason	CONDITION	Hypothetical
	Result		Factual
	Claim		Non-Factual
	Justification		

PURPOSE	Goal	TEMPORAL	Synchronous
	Enablement		Precedence
			Succession

CONCESSION	Contra-Expectation	ALTERNATIVE	Chosen-Alternative
	Expectation		Conjunctive
			Disjunctive

CONTRAST		INSTATIATION	

CONJUNCTION		EXCEPTION	

SIMILARITY		CONTINUATION	

CIRCUMSTANCE	Forward-Circumstance	BACKGROUND	Forward-Background
	Backward-Circumstance		Backward-Background

RESTATEMENT	Equivalence	REINFORCEMENT	
	Generalization		
	Specification		

For any relation, the sense annotation consists of selecting a sense subtype label whenever subtypes are available for a type. Thus, for the "Cause" sense, the annotator is required to select one of its four subtypes, i.e., the type level label cannot be chosen. Type-level labels can only be selected when the sense does not have any subtypes available, for example "Contrast". Refinements at the subtype level are of two kinds. One kind specifies refinements of the semantics, while the other kind specifies the directionality of the arguments. Thus, for example, the three subtypes of the "Condition" sense type specify in more detail the nature of the conditional dependence between Arg1 (antecedent) and Arg2 (consequence), by indicating whether the antecedent describes a hypothetical situation ("Hypothetical"), an assumed fact ("Factual"), or a non-fact ("Non-Factual"). On the other hand, the two subtypes of the "Concession" sense (in which one argument creates an expectation denied by the other argument) indicate the directionality of the concession: In the "Contra-expectation" subtype, Arg1 raises the expectation that Arg2 denies, while in the "Expectation" subtype, Arg2 raises the expectation that Arg1 denies.

With some connectives, more than one sense can be inferred. Annotators are allowed to assign upto two senses to a connective. In Example (17), for instance, two senses are annotated for the connective *as*: "Temporal:Synchronous" and "Cause:Justification".

(17) Tumors detected by this new technology could have unique etiologies and/or presentations, *and may represent an increasing proportion of clinical practice *as**new screening methods are validated and applied**. (Temporal:Synchronous/Cause:Justification)

The BioDRB sense classification was adapted from the PDTB sense classification [93]. Below, we first define the BioDRB senses, and then discuss the major differences with PDTB.

##### Cause

The sense type "Cause" is used when the two arguments of the relation are related causally and are not in a conditional relation. There are four subtypes for this sense. "Reason" and "Result" hold when the situation described in one of the arguments is the cause of the situation described in the other argument. They differ from each other only in the directionality of the causality. "Reason" is used when Arg2 is the cause and Arg1 the effect, while "Result" is used when Arg1 is the cause and Arg2 the effect. The other two subtypes, "Claim" and "Justification", hold when the situation described by one of the arguments is the cause, not for the situation described by the other argument, but rather for the truth or validity of the proposition described by the argument. The difference between the two is again in directionality, with "Claim" used when Arg1 presents the evidence for the truth of Arg2, and "Justification" used when Arg2 presents the evidence for the truth of Arg1.

##### Condition

The sense type "Condition" is used to describe all subtypes of conditional relations. There are three subtypes. The subtype "Hypothetical" holds when if Arg2 holds true, Arg1 is caused to hold at some instant in all possible futures. However, Arg1 can be true in the future independently of Arg2. The subtype "Factual" is a special case of the subtype "Hypothetical", and applies when Arg2 is a situation that has either been presented as a fact in the prior discourse or is believed by somebody other than the speaker/writer. The subtype "NonFactual" applies when Arg2 describes a condition that either does not hold at present or did not hold in the past. Arg1 then describes what would also hold if Arg2 were true. (There were no occurrences of the Non-Factual conditionals in the corpus.)

##### Purpose

The sense type "Purpose" is used when one argument presents a situation and the other argument presents an action, and the engagement of the action enables the situation to occur. The two subtypes "Goal" and "Enablement" capture difference in directionality: "Goal" applies when Arg1 presents an action that enables the situation in Arg2 to obtain, whereas "Enablement" applies when Arg2 presents an action that enables the situation in Arg1 to obtain.

##### Temporal

The sense type "Temporal" is used when the events described in the arguments are related temporally. There are three subtypes, which reflect the ordering of the arguments. "Precedence" is used when the Arg1 event precedes the Arg2 event; "Succession" applies when the Arg1 event follows the Arg2 event; and "Synchronous" applies when the Arg1 and Arg2 events overlap.

##### Concession

The sense type "Concession" applies when one of the arguments describes a situation A that creates an expectation for a situation C, while the other asserts (or implies) ¬C. Two "Concession" subtypes capture a difference in the roles of the arguments. "Expectation" is used when Arg2 creates an expectation that Arg1 denies, while "Contra-Expectation" is used when Arg1 creates an expectation that Arg2 denies.

##### Contrast

The sense type "Contrast" is used when the values for some shared property in Arg1 and Arg2 are in opposition to each other. These oppositions need not be at opposite ends of a graded scale and can be context-dependent. There are no subtypes for this sense.

##### Similarity

The sense type "Similarity" is like "Contrast" in that it involves the comparison of the values for some shared property of Arg1 and Arg2. The compared values in this case are similar to each other (and not in opposition).

##### Alternative

The sense type "Alternative" is used when the two arguments denote alternative situations. There are three subtypes. The "Conjunctive" subtype is used when both alternatives hold or are possible. The "Disjunctive" subtype is used when two situations are evoked in the discourse but only one of them holds. The "Chosen Alternative" subtype is used when multiple alternatives are evoked in the discourse, and one argument asserts that one of the alternatives was chosen.

##### Instantiation

The sense type "Instantiation" is used when Arg1 evokes a set and Arg2 instantiates one or more elements of the set. What is evoked may be a set of events, a set of reasons, or a generic set of events, behaviors, attitudes, etc. There are no subtypes for this sense.

##### Restatement

The sense type "Restatement" is used when the situation described by Arg2 restates the situation described by Arg1. The three subtypes "Specification", "Generalization", and "Equivalence" further specify the ways in which Arg2 restates Arg1. "Specification" applies when Arg2 describes the situation described in Arg1 in more detail. "Generalization" applies when Arg2 summarizes Arg1, or in some cases expresses a conclusion based on Arg1. "Equivalence" applies when Arg1 and Arg2 describe the same situation from different perspectives. (There are no occurrences of the "Equivalence" sense in the corpus.)

##### Conjunction

The sense type "Conjunction" is used when Arg1 and Arg2 are members of a list, defined in the prior discourse, explicitly or implicitly. No subtypes are defined for this sense.

##### Exception

The sense type "Exception" applies when Arg2 specifies an exception to the generalization specified by Arg1. In other words, Arg1 is false because Arg2 is true, but if Arg2 were false, Arg1 would be true. No subtypes are defined for this sense.

##### Reinforcement

The sense type "Reinforcement" is used when Arg2 is provided as fact to support claims or effects associated with Arg1. No subtypes are defined for this sense.

##### Continuation

The sense type "Continuation" applies when Arg1 expands the discourse by identifying an entity (concrete or abstract) in Arg1 and saying something about it. Crucially, for this relation, it must be the case that no other discourse relation holds. "Continuation" occurs frequently as an implicit relation, but it can also be associated with the explicit connective *and*.

##### Circumstance

The sense type "Circumstance" is used when one argument provides the circumstances under which the situation in the other argument was obtained. No causal relation is implied here. In BioDRB, this relation was introduced specifically to capture the circumstantial relation between an experimental set-up and the observations and results obtained from the experiments. Two subtypes capture difference in directionality. In "Backward Circumstance", Arg1 describes the circumstance and Arg2 describes the resulting situation. In "Forward Circumstance", Arg2 describes the circumstance and Arg1 describes the resulting situation.

##### Background

The sense type "Background" is used when one argument provides information that is deemed necessary or desirable for interpreting the other argument. Two subtypes capture difference in directionality. In "Backward Background" Arg1 provides the background information for Arg2, while in "Forward Background", Arg2 provides the background information for Arg1. No further subtypes are specified for this sense.

The BioDRB sense classification reflects the following changes from the PDTB classification:

• First, in the PDTB, the sense classification consists of three tiers, with four sense classes at the top tier. Three of the four class-level senses in the PDTB (namely, "Contingency", "Temporal", "Comparison", and "Expansion") are eliminated as we felt they were too broadly-defined to be useful. The only class-level sense we retained is "Temporal", but this has been reassigned as a type-level sense in the two-level BioDRB hierarchy.

• Second, we have collapsed some of the subtype-level senses. For the "Condition" sense type, for example, we do not maintain the PDTB distinction between the subtypes "Present-Factual" and "Past-Factual", and label both as "Factual". A similar reduction is done for "Non-Factual".

• Third, we have introduced some new senses, namely "Purpose", "Similarity", "Continuation", "Background", "Reinforcement". "Continuation" and "Background" are reformulations of the PDTB EntRel (Entity Relation) relation type, whereas "Purpose", "Similarity", and "Reinforcement" are senses that we believe were confounded with other senses in PDTB. For example, "Purpose" relations were annotated as "Result", "Similarity" relations were annotated as "Conjunction", and "Reinforcement" relations were annotated as either "Conjunction" or "Restatement".

• Finally, we have eliminated the separate type-level representation of pragmatic senses and have instead listed them as subtypes. These apply to the current subtypes for "Cause", namely "Claim" and "Justification". We did not find instances of the other pragmatic senses listed in PDTB.

Even though the PDTB class-level senses are not used in BioDRB, it is still possible to reconstruct the PDTB sense classes from the BioDRB sense types. This may be important for comparing the performance of NLP methods across the two domains, as we have needed to do for our own experiments on sense disambiguation below. Table 2 provides the reconstructed generalization of the BioDRB sense types into the four sense classes of PDTB.

**Table 2:** Grouping of BioDRB sense types into PDTB generalized classes

BioDRB Type-level Senses	PDTB Class-level Sense
Concession, Contrast	Comparison

Cause, Condition, Purpose	Contingency

Temporal	Temporal

Alternative, Background, Circumstance, Conjunction, Continuation, Exception, Instantiation, Reinforcement, Restatement, Similarity	Expansion

### Summary of BioDRB Annotations

The BioDRB corpus is available through the GENIA corpus release site http://www-tsujii.is.s.u-tokyo.ac.jp/GENIA/. BioDRB contains a total of 5859 relation tokens for the four different relation types: Explicit, Implicit, AltLex and NoRel. Table 3 shows the relation type distribution in the corpus. Token counts are given in the second column, and the unique (expression) types are shown in the third column. In counting the unique types for explicit connectives, we did not treat modified and unmodified connective expressions as the same type. Thus, for example, the connectives *after *and *one day after *were treated as distinct types. For implicit relations, we counted the connectives that were inserted by the annotators.

**Table 3:** BioDRB distribution of relation types

Relation Type	No. of Tokens (%)	Types
Explicit	2636 (45%)	179

Implicit	3001 (51.2%)	57

Altlex	193 (3.3%)	165

NoRel	29 (0.5%)	-

TOTAL	5859	-

Table 4 shows the sense distributions across the different relation types. Since explicit connectives and AltLex expressions can have multiple senses, we have listed multiple sense occurrences separately, to illustrate the extent of this kind of ambiguity. Note that for implicit relations, multiple senses are not permitted.

**Table 4:** Distribution of senses in BioDRB.

Sense	Explicit	Implicit	AltLex	TOTAL
Alternative	31	3	3	37

Background	-	132	1	133

Cause	339	98	105	542

Circumstance	8	221	1	230

Concession	257	70	2	329

Condition	22	-	-	22

Conjunction	421	641	3	1065

Continuation	24	831	-	855

Contrast	205	75	2	282

Exception	7	2	-	9

Instantiation	21	53	14	88

Purpose	616	-	1	617

Reinforcement	22	60	19	101

Restatement	69	445	19	533

Similarity	5	-	-	5

Temporal	394	370	16	780

				

Cause/Background	8	-	-	8

Cause/Conjunction	5	-	-	5

Cause/Reinforcement	-	-	1	1

Cause/Temporal	6	-	3	9

Concession/Background	2	-	-	2

Concession/Circumstance	1	-	-	1

Condition/Circumstance	2	-	-	2

Condition/Temporal	5	-	-	5

Conjunction/Temporal	70	-	1	71

Continuation/Reinforcement	1	-	-	1

Contrast/Background	-	-	1	1

Contrast/Concession	1	-	-	1

Purpose/Conjunction	1	-	-	1

Reinforcement/Conjunction	-	-	1	1

Temporal/Circumstance	92	-	-	92

Temporal/Continuation	1	-	-	1

**TOTAL**	2636	3001	193	5830

Multiple senses provided for connectives are shown separately.

In a given context, explicit connectives can have multiple sense interpretations, as shown in Table 4. However, a given connective can have different sense interpretations in different contexts as well. The extent of contextual ambiguity is shown in Table 5. For connectives with multiple senses, only the first sense provided in the annotation is used here. There are a total of 27 connectives types (column 1) exhibiting sense ambiguity to varying degrees.

**Table 5:** Contextual ambiguity of explicit connectives

Connective Type	Senses	Tokens
accordingly	2: Cause, Conjunction	2

although	2: Concession, Contrast	76

and	6: Cause, Concession, Conjunction, Continuation, Purpose, Temporal	274

as	3: Cause, Purpose, Temporal	23

both upon	2: Circumstance, Temporal	2

but	2: Concession, Contrast	42

by	3: Cause, Purpose, Temporal	262

nally	2: Conjunction, Temporal	21

however	2: Concession, Contrast	117

in part by	2: Cause, Purpose	3

in particular	2: Instantiation, Restatement	4

in response to	3: Cause, Circumstance, Temporal	12

in turn	3: Cause, Conjunction, Temporal	6

in	2: Circumstance, Purpose	3

indeed	2: Circumstance, Reinforcement	15

on the other hand	2: Concession, Contrast	6

once	2: Circumstance, Temporal	7

second	2: Conjunction, Temporal	3

since	2: Cause, Temporal	52

so	2: Cause, Restatement	7

then	2: Restatement, Temporal	91

therefore	2: Cause, Restatement	75

thus	2: Cause, Restatement	77

upon	2: Cirsumstance, Temporal	15

when	3: Circumstance, Condition, Temporal	65

while	4: Concession, Conjunction, Contrast, Temporal	64

whilst	2: Concession, Contrast	4

Total	-	1328

Column 2 provides the number and names of different senses associated with the connectives, while column 3 provides the total number of tokens for the connective. The total number of tokens for all these ambiguous connectives is 1328, which constitutes 50.4% (1328/2636) of the total number of explicit connective tokens.

### Annotation Task Procedure

For the task of annotating discourse relations, each annotator was given an article and instructed to read the article from beginning to end while marking up relations. No pre-defined lists of connectives were provided to annotators, although the connective list from PDTB was provided as an example of what to look for. Annotators were strongly encouraged to identify additional connectives when they were observed. At a high-level, the annotation procedure is encapsulated as follows:

For every new sentence encountered while reading the text:

1. First determine if there is an explicit connective that relates the sentence to the prior context via a discourse relation. If so, mark this explicit connective, its arguments, and its sense(s). Label the relation type as *Explicit*.

2. If there is no explicit connective present to relate the sentence with the prior context, try to insert an implicit connective to express the inferred implicit relation, annotate its sense, and mark its arguments. In case the inferred relation is one of the senses of "Continuation", "Background", or "Circumstance", no connective can be inserted, so use the dummy label "NONE" in place of an implicit connective. Label the relation type as *Implicit*.

3. If insertion of an implicit connective leads to redundancy in the expression of the relation, identify and mark the AltLex expression that expresses the relation, annotate its sense, and mark its arguments. Label the relation type as *AltLex*.

4. If the sentence does not seem to relate coherently to any sentence in the prior text, label the relation type as *NoRel*, mark the current sentence as Arg2 and the previous sentence as Arg1.

5. After annotating the relation of the sentence with the previous context, identify and annotate any sentence-internal explicit connectives that have both their arguments in the same sentence.

### Limitations

While we believe that the scope of discourse relations captured in BioDRB is larger than that of the framework from which it was adapted, there are two types of relations that are currently not handled. We describe these below. The main reason for their exclusion is the challenge associated with their annotation. We plan to address these challenges in future extensions to the corpus.

First, we have not annotated implicit or AltLex relations between events and situations mentioned within a single sentence. For example, in the sentence "In particular, after binding with E2, oestrogen receptors have been shown to interact with NF-*κ*B factors, via transcriptional co-factors, resulting in mutual or non-mutual antagonism.", an Altlex "Result" relation can be inferred between the "interaction of oestrogen receptors with NF-*κ*B factors" and "mutual or non-mutual antagonism", anchored in the verb *resulting*. Such relations were excluded because it is challenging to identify the clausal boundary "sites" where they are inferred. Although the syntactic parse of a sentence can be used for this purpose, we did not have a sufficiently accurate sentence parser for our texts.

Second, coordinating conjunctions (e.g., *and, or*) that conjoin verb phrases in a sentence can potentially indicate discourse relations between two situations. What's more, the conjunction *and *can often express more than the sense of "Conjunction", including at least the "Temporal" and "Result" senses. For example, the cojunction *and *in the sentence "Thus SEB can interact directly with MHC class II molecules on APCs and activate T cells bearing the proper TcR V*β *chains." can be taken to express a conjunction of two independent situations, namely "SEB interacting with MHC class II molecules on APCs" and "SEB activating T cells bearing the proper TcR V*β *chains". In addition, either a causal, temporal or enablement relation might be inferred here. While such conjunctions appear often in the BioDRB, we decided to exclude them because it is difficult to distinguish them from conjunctions that don't have a discourse function.

### Evaluation of Annotation Reliability

Each article was annotated by two annotators who were premed students at the University of Pennsylvania. The domain expertise of the annotators is crucial for allowing them to identify the correct sense of discourse connectives and to identify the existence of implicit relations. The annotators were extensively trained (by the first author) with regard to knowledge of linguistic syntax, semantics, and discourse, following which they were given a tutorial on the biomedical discourse annotation guidelines. The annotation was carried out over a period of three years, with annotators annotating at an average speed of 7 minutes per relation.

We computed agreement for connective identification, argument identification and sense labeling. Explicit and AltLex relations were treated separately from implicit relations.

For agreement on the identification of explicit connectives and AltLex expressions, we calculated the percentage of overlapping tokens identified by the annotators, since one annotator could have selected some connectives or AltLex's that the other did not. For example, if one annotator identified 20 connectives and the other identified 30 connectives, this could mean that there were 15 tokens that were common to both, and that there were 35 tokens some of which were identified by one annotator while the others were identified by the other annotator. The agreement was then reported as the percentage of common over common and uncommon tokens (i.e., 43% (15/35) for the artificial case illustrated above). We achieved 82% agreement. The major sources of mismatch were subordinators, which are harder to identify than conjunctions and adverbials, and AltLex's.

For agreement on argument spans, we used both the exact match criterion as well as the more relaxed partial match criterion [73]. With the exact match criterion, annotators are taken to agree on an argument only when their respective selections are identical or fully overlapping, whereas the partial match criterion allows agreement even in the case of partial overlap. Argument agreement was computed only on the connectives where the annotators agreed. For Explicit and AltLex relations, we achieved an exact match of 88% and 81% on Arg2 and Arg1, respectively. This difference is understandable, since Arg1s are generally harder to identify than Arg2s. With partial match, we achieved an agreement of 93% and 86% for Arg2 and Arg1, respectively. Agreement on implicit relations was lower, at 88% and 75% for Arg2 and Arg1, respectively. The most likely reason for lower agreement for implicits is that non-adjacent arguments were allowed in the BioDRB, which makes the task of identifying the arguments harder.

Since sense guidelines allow an annotator to select multiple senses for a given connective, we took annotators to agree on sense labeling if at least one sense for a connective was the same across both annotators. Furthermore, since the sense labeling task involved classifying a given set of connectives into multiple nominal categories, namely 31 sense categories in total (see Table 1), we report the agreement by computing the kappa score. For explicit and AltLex relations, the kappa score was 0.71, with the observed agreement at 0.85 and the expected agreement at 0.48. For implicit relations, the kappa score was 0.63, with the observed agreement at 0.82 and the expected agreement at 0.52. The kappa scores for both explicit and implicit relations are therefore in the range generally accepted as substantial agreement.

Following the double-blind annotation and agreement calculations, the disagreements were adjudicated by an expert. We also made further reviews of the corpus to correct for any remaining guideline-related errors.

### BioDRB Data, Tools and Representation

#### Data

The source corpus over which the BioDRB has been annotated consists of 24 full-text articles from the GENIA corpus [39]. The GENIA corpus is a collection of articles from the biomedical literature. It has been compiled and annotated within the scope of the GENIA project http://www-tsujii.is.s.u-tokyo.ac.jp/GENIA/.

The 24 GENIA articles were selected by the GENIA group in 2006 by searching the PubMed entries with two MeSH terms "Blood cells" and "Transcription factors". Among the returned entries, 24 articles were open-access that are considered representative of the scientific text style of this domain [94]. This full-text data collection has been annotated with coreference (by the GENIA group) and citation relations [61], and therefore represents one of the most comprehensively annotated full-text biomedical corpora. Our annotation of discourse relations on this corpus will further enrich the data resource, and will assist future text mining applications.

Altogether, the articles have a total of 112483 words and 4911 sentences. Sentence counts were obtained with the UIUC sentence segmentation tool http://cogcomp.cs.illinois.edu/page/tools_view/2.

#### Annotation Tools and Representation

We used a recently released version of the discourse annotation tool, called "Annotator", distributed by the PDTB group. It is freely available from http://www.seas.upenn.edu/~pdtb/PDTBAPI, and differs from earlier versions primarily with respect to its simpler data representation. The tool allows for the annotation of relations, their arguments, as well as senses, all within the same interface.

Following PDTB, BioDRB annotations are represented in a "stand-off" style, in that the annotation files are physically distinct from the source files. Text span annotations are represented in terms of their character o sets in the source files, and can be easily retrieved programmatically. When text spans are discontinuous, which is possible for both connective spans and argument spans, they are represented as sets of offsets. Each element of the set is associated with one part of the discontinuous spans and the order of the elements in the set reflects the linear order of the discontinuous spans in the text. Annotation files are at text files, with each line representing a single relation token and all its annotated features (separated with the " |" delimiter). Since we used the tool developed initially for PDTB, which also annotated additional attribution features, only some of the "|" separated fields are relevant for BioDRB. These are shown in Table 6. The first column provides the field number (starting count from 0) and the second column describes the annotation that the field contains. Other fields are simply left blank. For implicit relations, no span offsets are provided since there is no lexical item associated with the relation. To identify the location of the implicit relation, the start offset of its Arg2 span is used as the identifier.

**Table 6:** Annotation fields in the BioDRB data representation

**Field Num**.	Description
0	Relation type (Explicit, Implicit, AltLex, NoRel)

1	(Sets of) Span o sets for connective (when explicit)

7	Connective string "inserted" for Implicit relation

8	Sense1 of Explicit Connective (or Implicit Connective)

9	Sense2 of Explicit Connective (or Implicit Connective)

14	(Sets of) Span o sets for Arg1

20	(Sets of) Span o sets for Arg2

Table 7 shows several examples of the annotation representation. Row 1 shows the entry of multiple senses ("Temporal.Precedence" and "Conjunction") for an explicit connective. Row 2 shows a set of span o sets ("21670..21678;21729..21737") for a discontinuous explicit connective text span, with the elements separated by a semi-colon. A discontinuous text span for Arg2 ("10090..10100;10106..10209") is shown in Row 3. Rows 4 and 5 show the annotation for an implicit and AltLex relation, respectively.

**Table 7:** Annotation representation

Explicit|9171..9174|||||||Temporal.PrecedencejConjunction|||||9137..9170||||||9175..9244||||||
Explicit|21670..21678;21729..21737|||||||Conjunction||||||21679..21727||||||21738..21829||||||

Explicit|10101..10105||||||||Temporal.Precedence||||||9932..10088||||||10090..10100;10106..10209||||||

Implicit||||||||as a resultjCause.Result||||||3418..3655||||||3657..3714||||||

AltLex|25183..25199||||||||ReinforcementjCause.Claim||||||24621..25181||||||25183..25444|||||||

### Sense Detection of Explicit Connectives

Predicting the sense of discourse relations is an important subtask of discourse parsing. Prior work on discourse relation sense detection has tackled the task of identifying the senses of explicit connectives separately from implicit relations. Sense prediction for explicit connectives in the open-domain PDTB has been shown to be an easy task, with most connectives being unambiguous [44,52]. As a result, the connectives themselves serve as highly reliable predictors of their sense.

In this section, we describe our preliminary experiments for classifying the senses of explicit connectives in BioDRB. Similar to prior work with the PDTB, one of our goals here is to establish a baseline for this task by using just the (case-insensitive) connective text string as the predictive feature. We also carried out the same experiments with the PDTB data, in order to compare the results across the two domains, as well as to explore how well a classifier trained on the open-domain PDTB data generalizes to the domain-specific data of the BioDRB (described in the next section). For all experiments, we used SLIPPER [95], a learning system that generates rulesets based on confidence-rated boosting.

To effectively compare BioDRB and PDTB, we need to group the BioDRB sense types into the 4 generalized classes in the PDTB (Table 2), and perform 4-way classification for these generalized senses. The main reason for designing the comparative study at the class-level instead of the type-level is that sense annotation in the PDTB follows a " flexible" approach, wherein annotators are allowed to back-o to the most general class-level in the hierarchical classification. As a result, many connectives in PDTB are labeled with only class-level senses, which makes their comparison difficult with the type-level senses in BioDRB.

Since explicit connectives can have up to two senses (see Table 4), we allowed for three scenarios. In the first scenario, only the *first sense *of a connective was considered, yielding a total of 2636 sense instances. In the second scenario, only the *second sense *was considered. There are 195 such instances (7.4%) in the BioDRB. Selecting the second sense also yielded a total of 2636 sense instances. Finally, in the third scenario, we allowed for *both senses *to be selected, so that the data set consists of new sense instances for the 195 multiple-sense connectives. This yielded a total of 2831 (2636+195) sense instances. Our hypothesis was that the third scenario increases sense ambiguity in the data, and that the classifier performance should therefore decrease.

For the PDTB experiments, we used the same data set used in other previous work, and considered the same three scenarios described above for connectives with two senses. Of the 18459 explicit connectives in PDTB, 999 (5.4%) appear with two senses.

In all cases, we carried out ten-fold cross-validation. For BioDRB, the majority class was the "Contingency" sense, giving a baseline of 35%, averaging across all three scenarios. Average baseline for PDTB was 33%, with "Expansion" as the majority class. Results are reported in Table 8, showing that the overall classification performance is very similar across the two corpora. (Note that other previous work with the PDTB has been done for the third *both sense *scenario [44,52], where a higher accuracy of 93% is reported. However, Pitler et al. used a Naive Bayes classifier in their experiments, and we expect that such a classifier on the BioDRB data would perform at similar levels.) Thus, explicit sense prediction can be done very reliably in the biomedical domain as well, using the connective as the only predictive variable. Also, the fact that the performance degrades when both senses of a multi-sense connective are considered confirms our hypothesis that this scenario increases ambiguity in the data. However, it is interesting to find that in both corpora, the performance is lowest when only the second sense is considered. It is possible that the second senses that were provided by annotators are often weak interpretations of the discourse relation, and that the first sense is the stronger, preferred, interpretation.

**Table 8:** Ten-fold cross validation accuracies for explicit connective sense classification in BioDRB and PDTB.

	First Sense	Second Sense	Both Senses
**BioDRB**	90.9%	83.6%	85.6%
**PDTB**	90.1%	84.1%	85.6%

Columns represent three scenarios for selecting from multiple senses provided for connectives.

In all remaining experiments here, we use the data from the *first sense *scenario, for which the classifier performs best. Macro average F1 score for both corpora was 0.91.

To examine how the classifier performs on each of the different classes, we computed the class-wise precision, recall and F1 score. The results in Table 9 show that the worst scores are precision for "Contingency" (0.82) and recall for "Temporal" (0.75). Interestingly, a similar experiment with the PDTB (results shown in Table 10) shows the same two senses with the worst scores, but here, it is recall for "Contingency" (0.71) and precision for "Temporal" (0.88). This suggests that there might be some differences in the semantic usage of connectives across the two domains.

**Table 9:** Explicit sense classification in BioDRB: Class-wise Precision, Recall and F1.

Class	Precision	Recall	F1
Comparison	0.983	0.868	0.922
Contingency	0.819	0.992	0.897
Expansion	0.923	0.9	0.911
Temporal	1.0	0.754	0.860

Macro average F1 score is 0.91.

**Table 10:** Explicit sense classification in PDTB: Class-wise Precision, Recall and F1.

Class	Precision	Recall	F1
Comparison	0.948	0.993	0.970
Contingency	1.0	0.706	0.828
Expansion	0.907	0.978	0.941
Temporal	0.883	0.889	0.886

Macro average F1 score is 0.91.

Next, we considered whether the size of the BioDRB corpus is sufficient for sense detection. Given that the accuracy of the BioDRB classi er is at the same level as that trained on the more than 8 times larger PDTB, this suggests that the BioDRB corpus size may be sufficient for this task. We tested our conjecture by partitioning the data into a training set (2360 instances) and test set (276 instances), and incrementally increasing the size of the training examples, in order to see if the classifier performance stabilizes as the training size reaches the maximum, *n *= 2360. We used 8 increments (236 examples in each increment), using the same test set of 276 examples with each incremented training set. The results show that the peformance of the classifier improves up to *n *= 1888, achieving an accuracy of 90.6%, but further increments up to *n *= 2360 do not significantly improve the performance. We therefore conclude that the size of the BioDRB corpus is sufficient for the task of explicit connective sense identification. Furthermore, these results are consistent with our related work on connective identification in BioDRB [45], where we show that the performance of the classifier becomes stable when the training size reaches over 5000 words.

Finally, since the BioDRB sense classification was designed to provide more refined and, therefore, more informative sense distinctions, we performed classification with the 15 type-level senses for explicit connectives. (Note that the 16th sense, "Background", does not appear for explicit connectives.)

The majority class (the "Purpose" sense) baseline accuracy for the type-level senses was 23.5%. Again, we performed a ten-fold cross-validation on the full data set of 2636 connectives, considering only the first sense of the connective where multiple senses were provided. Not surprisingly, the accuracy of the classifier for more refined classification is lower, at 69.2%, although still significantly higher than the baseline. The macro average F1 score was 0.28, mainly because many senses are too sparse for rules to be learned reliably. Examination of class-wise scores shows that rules were reliably learned for three senses - "Temporal" (F1 score 0.94), "Conjunction" (F1 score 0.97), "Cause" (F1 score 0.81) - all of which have more than 300 instances each in the corpus (see Table 4). While these results suggest that we may need more annotated training data for reliable refined sense classification, our immediate goal is to first explore the use of richer features for the classifier. We conjecture that for more refined sense classification, the connective is not sufficient as the sole predictive variable.

### Lessons to be Learned from a New Domain

A natural question that arises in the context of our work is whether it is necessary to develop an independently annotated biomedical corpus of discourse relations, instead of using tools that have already been developed for the open domain. In this section, we present two studies showing that developing an independent domain-specific corpus is indeed beneficial. Our conclusions are consistent with *sublanguage theories *[96-98] for technical domains such as the biomedical domain.

First, as demontrated in the previous section, although BioDRB and PDTB sense classifiers perform at very similar levels of accuracy, there are class-wise differences in performance which suggest differences in the semantic usage of connectives across the two domains. To explore this further, we trained the classifier on the PDTB data and tested it on BioDRB. The accuracy of this cross-domain classifier was 54.5% and the macro average F1 score was 0.57. Class-wise precision, recall and F1 scores reported in Table 11 show that "Comparison" is the only sense with scores comparable to the within-domain classifier (see Table 9), with all other senses performing much worse. These results indicate that a sense classifier trained on the open-domain PDTB data does not generalize well to the biomedical domain, and that there is a significant advantage to developing an independent biomedical annotated corpus of discourse relations. Our findings here are consistent with our related work on identifying connectives in BioDRB [45], which shows that a connective identification classifier trained on PDTB does not perform well on BioDRB even with domain adaptation techniques (instance weighting, instance pruning, and feature augmentation), compared to a classifier trained on the BioDRB alone.

**Table 11:** Cross-domain sense classification: Class-wise Precision, Recall and F1.

Class	Precision	Recall	F1
Comparison	0.983	0.897	0.938
Contingency	0.643	0.732	0.131
Expansion	0.347	0.938	0.507
Temporal	0.863	0.585	0.697

Macro average F1 score is 0.57.

Second, given that texts from the biomedical literature are typically segmented into the rhetorical categories of *Introduction, Methods, Results and Discussion *(IMRAD) [99-102], we explored whether discourse relations within each of these segments exhibit regular patterns.

We examined all relation types (i.e, explicit, implicit, and Altlex) when they appeared in the clearly indicated IMRAD segments. Relations in other sections were ignored. For example, some articles did not have the conventional IMRAD structure at all, and were therefore ignored completely in our calculations. Further, sections such as *Conclusions, Authors' Contributions*, and *Figures and Table Captions *were ignored. Finally, in some cases, differently named sections were treated as the same. For example, *Background *sections were counted together with *Introduction*, and *Materials and Methods *were counted together with sections named *Methods*. In this way, we extracted the sense distribution for a total of 3953 explicit, implicit and AltLex relations for IMRAD segments, shown in Table 12.

**Table 12:** Sense distributions in IMRAD segments

Type-level Sense	Introduction	Methods	Results	Abstract	Discussion	Total
Alternative	4 (13.8%)	3 (10.3%)	7 (24.1%)	0 (0.0%)	15 (51.7%)	29
Background	24 (19.8%)	7 (5.8%)	36 (29.8%)	15 (12.4%)	39 (32.2%)	121
Cause	80 (17.0%)	16 (3.4%)	134 (28.5%)	33 (7.0%)	208 (44.2%)	471
Circumstance	11 (7.1%)	7 (4.5%)	112 (71.8%)	13 (8.3%)	13 (8.3%)	156
Concession	59 (21.7%)	3 (1.1%)	73 (26.8%)	21 (7.7%)	116 (42.6%)	272
Condition	1 (5.3%)	6 (31.6%)	0 0 (0.0%)	1 (5.3%)	11 (57.9%)	19
Conjunction	105 (13.9%)	100 (13.3%)	271 (35.9%)	78 (10.3%)	195 (25.9%)	754
Continuation	80 (19.3%)	121 (29.2%)	112 (27.0%)	17 (4.1%)	85 (20.5%)	415
Contrast	26 (10.6%)	9 (3.7%)	118 (48.0%)	12 (4.9%)	81 (32.9%)	246
Exception	1 (16.7%)	2 (33.3%)	2 (33.3%)	0 (0.0%)	1 (16.7%)	6
Instantiation	17 (23.9%)	0 (0.0%)	9 (12.7%)	3 (4.2%)	42 (59.2%)	71
Purpose	93 (20.2%)	84 (18.3%)	144 (31.3%)	35 (7.6%)	104 (22.6%)	460
Reinforcement	14 (16.5%)	3 (3.5%)	14 (16.5%)	4 (4.7%)	50 (58.8%)	85
Restatement	63 (19.2%)	47 (14.3%)	124 (37.8%)	29 (8.8%)	65 (19.8%)	328
Similarity	0 (0.0%)	0 (0.0%)	2 (40%)	0 (0.0%)	3 (60%)	5
Temporal	41 (8.0%)	259 (50.3%)	0 (0.0%)	22 (4.3%)	52 (10.1%)	515

It is revealing to see that the *Methods *segments contain "Temporal" relations more frequently than the other segments, since these segments describe the various steps of experiments that have been conducted. The segments from *Methods *also have negligible "Concession" relations, suggesting that these sections lack reasoning or argumentation. Indeed, "Contrast" and "Concession" relations are found more frequently in the *Results *and *Discussion *segments, where comparisons are made with related work, and arguments are made about the presented work. Also frequent in the *Discussion *section are "Causal", "Instantiation", and "Reinforcement" relations, since authors give justifications, reasons, and, in general, reinforcing arguments for their experiments and conclusions. There is a high proportion of "Circumstance" relations in the *Results *section, where outcomes of experiments are presented. "Background" relations are, curiously, not more frequent in the *Abstract *and *Introduction *sections, as one would expect, but rather in the *Result *and *Discussion *section. Overall, these senses show several useful patterns in the distribution of senses across the different IMRAD segments, suggesting that biomedical literature contains a highly domain-specific distribution of relations that can benefit text-mining applications. In future work, we plan to explore the feasibility of using the IMRAD segment type as a feature for classifying the senses of explicit connectives.

## Conclusion

We have developed the Biomedical Discourse Relation Bank (BioDRB), which contains discourse-level annotations of explicit and implicit discourse relations and their abstract object arguments, and the senses of discourse relations. Starting with the Penn Discourse Treebank (PDTB) as the underlying discourse annotation framework because of its theory-neutral and lexically grounded approach, we have successfully adapted the PDTB annotation guidelines for the biomedical discourse annotation, while introducing some features specific to, and necessary for, the biomedical domain. We have also carried out experiments on sense detection of explicit connectives. Our results show that using the connective as the only feature for the classification creates a very high baseline for the task, as in the open domain. At the same time, there are significant differences in the semantic usage of connectives across the two domains, since a sense classifier trained on the PDTB data does not generalize to the BioDRB. Together with similar results that we have obtained in our related work on identifying explicit connectives, we conclude that it is beneficial to take a "sublanguage" approach for discourse processing of biomedical literature, and develop an independent biomedical corpus of discourse annotations. Finally, we have also found that while the size of the BioDRB corpus is sufficient for coarse-sense classification, more training data might be needed for more refined sense classification, although future research should first explore the use of richer features. One such additional feature may be the IMRAD segments of these articles, which show some useful patterns of sense distributions.

## Availability and Requirements

**Project name: **Biomedical Discourse Relation Bank Project

**Project home page: **http://www.biodiscourserelation.org

**Operating system(s): **Platform independent

**Programming language: **None

**Other requirements: **Java 1.5 or higher (for annotation tools)

**License: **None

**Any restrictions to use by non-academics: **None

## Authors' contributions

RP designed and directed the development of the BioDRB corpus, carried out all experiments, and drafted the manuscript. SM participated in contributed to the development of the annotation guidelines, and provided critical intellectual content for revisions on the draft. NF participated in the pilot annotation study and contributed to the development of the annotation guidelines. AJ contributed to the comparative studies in this work. HY conceived of the study and participated in its design and coordination. All authors have read and approved the final manuscript.
